# A Case of Recurrent Acute Pancreatitis After Gemcitabine + Nab-Paclitaxel and Modified FOLFIRINOX Therapy for Advanced Recurrent Pancreatic Cancer

**DOI:** 10.7759/cureus.102865

**Published:** 2026-02-02

**Authors:** Yusuke Inada, Haruna Sango, Noriki Kasuga, Hiroko Yukawa, Motohiko Sano

**Affiliations:** 1 Pharmacy Department, Yokohama Rosai Hospital, Yokohama, JPN; 2 Gastroenterology Department, Yokohama Rosai Hospital, Yokohama, JPN; 3 Oncology Department, Yokohama Rosai Hospital, Yokohama, JPN; 4 Laboratory of Clinical Pharmacy Assessment, Hoshi University, Tokyo, JPN

**Keywords:** chemotherapy, drug-induced pancreatitis, gemcitabine plus nab-paclitaxel therapy, modified folfirinox, pancreatic cancer

## Abstract

The incidence of drug-induced acute pancreatitis is an important consideration when developing treatment strategies for patients with pancreatic ductal adenocarcinoma. We report a case of recurrent acute pancreatitis that developed following both gemcitabine plus nab-paclitaxel (GnP) chemotherapy and modified FOLFIRINOX (mFFX) therapy. The clinical stage at initial diagnosis was cT3N1M0 stage IIB pancreatic cancer, with subsequent progression to metastatic disease over approximately 1.5 years. Treatment was prioritized at each stage of disease progression despite the occurrence of acute pancreatitis. Recurrent episodes of acute pancreatitis prompted a change in chemotherapy regimen from GnP to mFFX; however, a single causative agent could not be identified. For both chemotherapy regimens, the onset of acute pancreatitis occurred within 24 hours of treatment initiation, and the clinical course was mild, consistent with previous reports of drug-induced pancreatitis. Agents common to both regimens, specifically 5-hydroxytryptamine type 3 (5-HT3) receptor antagonists, may have contributed to the development of pancreatitis. Further accumulation of similar cases is required to better clarify the relationship between pancreatic cancer chemotherapy and the risk of acute pancreatitis.

## Introduction

Acute pancreatitis typically presents with abdominal pain and tenderness in the upper abdomen. In Japan, alcohol consumption and gallstones are the two most common causes of acute pancreatitis [1]. However, more than 500 drugs have been reported to cause acute pancreatitis as an adverse effect, including anticancer agents [2]. Although drug-induced acute pancreatitis is relatively rare, it can be severe or fatal; therefore, careful attention to this potential adverse effect is required [3].

Pancreatic ductal adenocarcinoma (PDAC) is one of the leading causes of cancer-related mortality worldwide, with a 5-year survival rate of approximately 10% [4]. At diagnosis, approximately 80% of patients present with locally advanced or metastatic disease and are not candidates for surgical resection [4]. Consequently, systemic chemotherapy is the standard initial treatment for advanced PDAC. Currently, first-line chemotherapy regimens for unresectable or metastatic PDAC include gemcitabine plus nab-paclitaxel (GnP) and FOLFIRINOX.

The GnP regimen consists of nab-paclitaxel (125 mg/m²) followed by gemcitabine (1,000 mg/m²), administered on days 1, 8, and 15 of a 28-day cycle [5]. The standard FOLFIRINOX regimen consists of oxaliplatin (85 mg/m²), irinotecan (180 mg/m²), leucovorin (400 mg/m²), followed by bolus 5-fluorouracil (400 mg/m²) and continuous infusion of 5-fluorouracil (2,400 mg/m²) every two weeks [6]. A modified FOLFIRINOX regimen (mFFX) has been developed to improve tolerability while maintaining efficacy [7]. In Japan, a commonly used modification omits the 5-fluorouracil bolus and reduces the dose of irinotecan to 150 mg/m² [8].

Despite the widespread use of these regimens, the risk of drug-induced acute pancreatitis has not been clearly established. Acute pancreatitis is not listed as an adverse effect in the package insert for gemcitabine (Gemzar®), and the reported incidence for nab-paclitaxel (Abraxane®) is less than 0.1%. Although the package insert for 5-fluorouracil mentions acute pancreatitis as a potential adverse effect, its incidence is unknown. The time course and recurrence pattern of pancreatitis following chemotherapy initiation remain poorly described.

Here, we report a case of unresectable pancreatic cancer in which acute pancreatitis occurred repeatedly within hours after initiation of GnP therapy, with recurrence even after switching to mFFX.

## Case presentation

A 60-year-old man was admitted to our hospital in April 2023 with obstructive jaundice. He had no significant medical or family history. His social history included alcohol consumption (approximately 350 mL of beer per day) and smoking (20 cigarettes per day), both of which he discontinued at the time of diagnosis. He had a documented allergy manifesting as a skin rash following intravenous administration of ampicillin/sulbactam.

**Figure 1:**
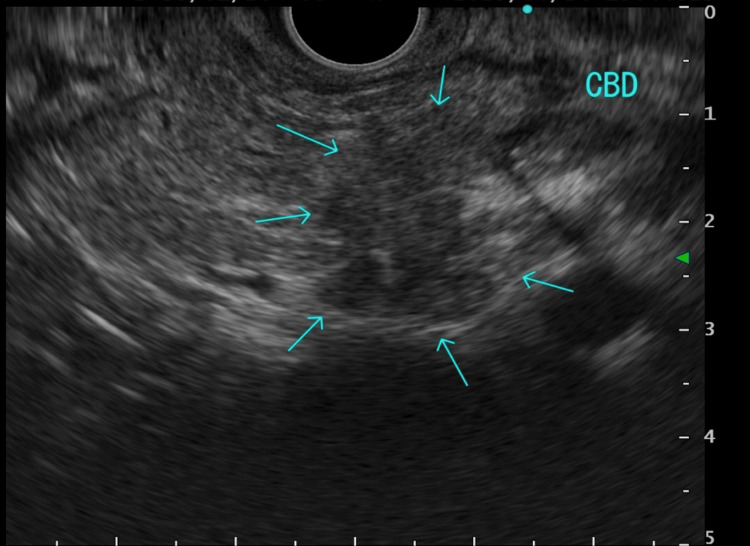
Endoscopic ultrasound of the pancreatic head (April 2023) Endoscopic ultrasound image of the pancreatic head obtained on April 24, 2023, showing a hypoechoic area with ill-defined margins and heterogeneous echotexture (arrows). No dilation of the common bile duct was observed.

Endoscopic ultrasound-guided fine-needle aspiration revealed a pancreatic head tumor, and the disease was staged as cT3N1M0 (stage IIB) pancreatic ductal adenocarcinoma (Figure 1). Preoperative chemotherapy with gemcitabine and S-1 was initiated but discontinued because of infectious complications, and subtotal pancreaticoduodenectomy was performed in July 2023, followed by adjuvant chemotherapy with S-1.

**Figure 2:**
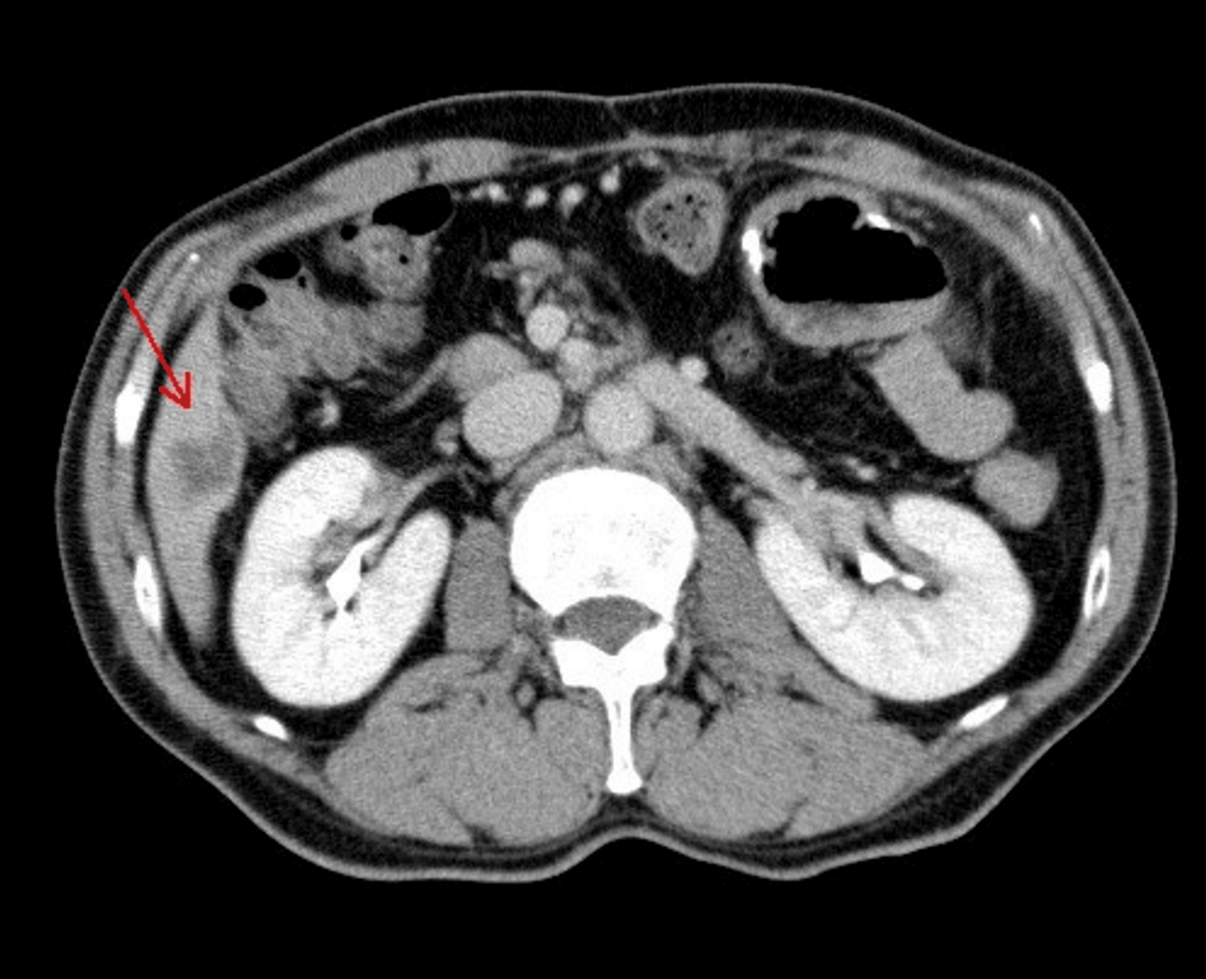
Contrast‑enhanced computed tomography prior to chemotherapy (February 2024) Contrast-enhanced computed tomography performed in February 2024, showing newly developed metastatic lesions in the liver (arrows) after pancreaticoduodenectomy.

In February 2024, serum CA19-9 levels increased markedly, and contrast-enhanced computed tomography (CT) revealed a hypoattenuating lesion measuring approximately 2.0 cm in hepatic segment 6, consistent with liver metastasis (Figure 2). The patient was diagnosed with recurrent pancreatic cancer, and GnP therapy was initiated on March 26, 2024. As antiemetic supportive care, intravenous granisetron (3 mg) and dexamethasone (6.6 mg) were administered immediately prior to chemotherapy.

**Figure 3:**
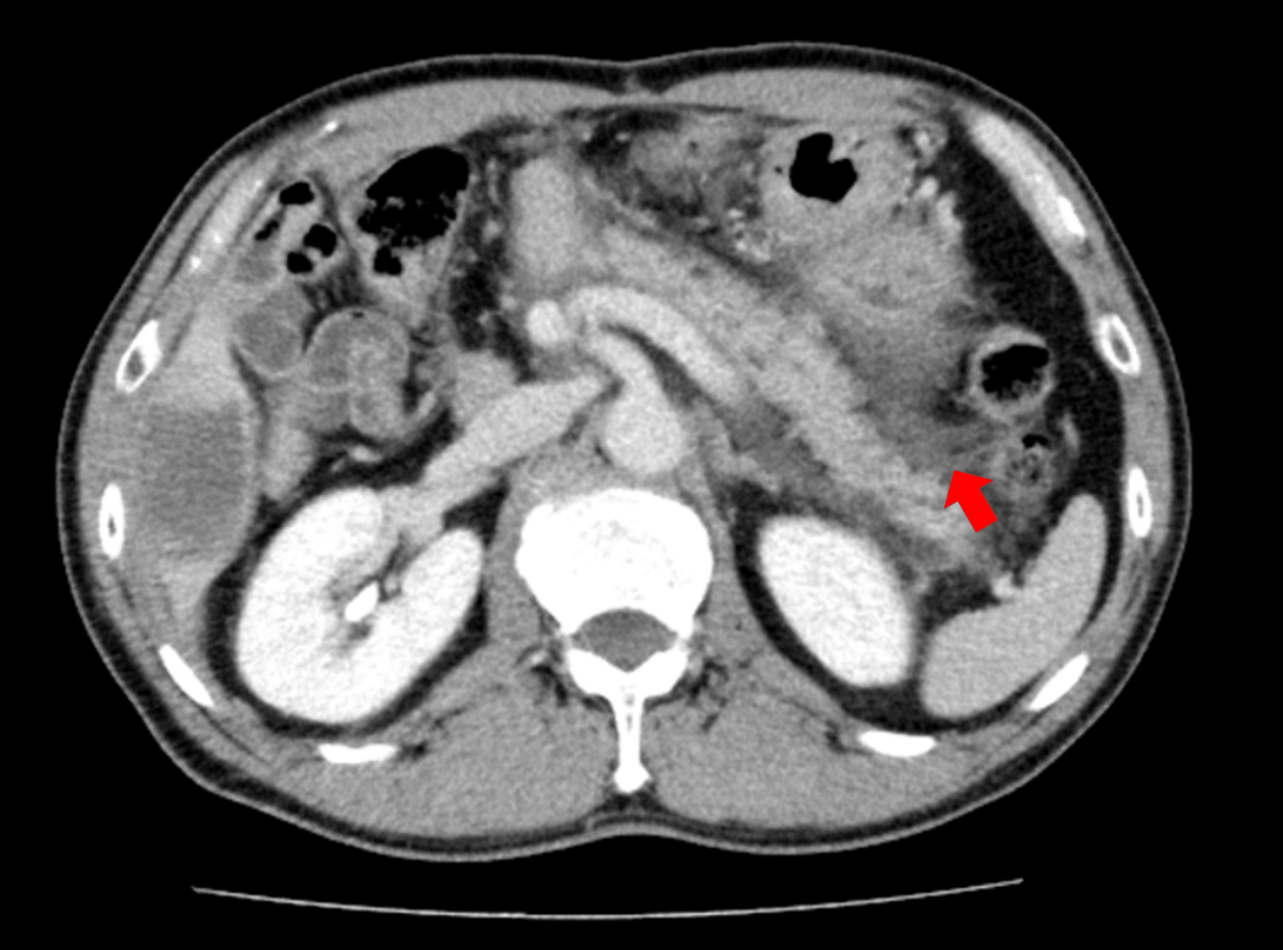
Contrast‑enhanced computed tomography at the first episode of acute pancreatitis (March 2024) Contrast‑enhanced computed tomography obtained in March 2024 during the first episode of acute pancreatitis shows inflammatory changes and peripancreatic fat stranding consistent with acute pancreatitis.

The patient returned home without symptoms; however, approximately 10 hours later, he developed acute-onset epigastric pain. Laboratory testing revealed marked elevation of pancreatic enzymes, and contrast-enhanced CT demonstrated inflammatory changes around the remnant pancreas without necrosis or fluid collection, consistent with mild acute pancreatitis (Figure 3). Laboratory findings at admission are summarized in Table 1.

**Table 1:** Laboratory findings at the onset of acute pancreatitis

Parameter	Result	Normal Value
White Blood Cell	10.0	3.3-8.6×103/µL
Hemoglobin	15.3	13.7-16.8g/dL
Platelet	258	158-348×103/µL
Sodium	141	138-145mEq/L
Potassium	4.2	3.6-4.8mEq/L
Chloride	101	101-108mEq/L
Calcium	9.3	8.8-10.1mg/dL
Blood Urea Nitrogen	15.5	8-20mg/dL
Creatinine	0.74	0.65-1.07mg/dL
Aspartate Aminotransferase	41	13-30U/L
Alanine Aminotransferase	32	10-42U/L
Total Bilirubin	0.9	0.2-1.2mg/dL
γ-Glutamyl Transpeptidase	51	13-64U/L
Creatine Kinase	68	59-248U/L
Total Protein	7.9	6.7-8.3g/dL
Albumin	4.7	4.1-5.1g/dL
Amylase	1,523	44-132U/L
Lipase	4,181	17-57U/L
C-reactive Protein	0.46	0-0.1mg/dL

The patient was treated conservatively with fasting, aggressive intravenous fluid therapy, and ulinastatin, resulting in rapid improvement. Endoscopic retrograde cholangiopancreatography was performed, and dilation of the pancreaticojejunostomy was carried out without stent placement.

**Figure 4:**
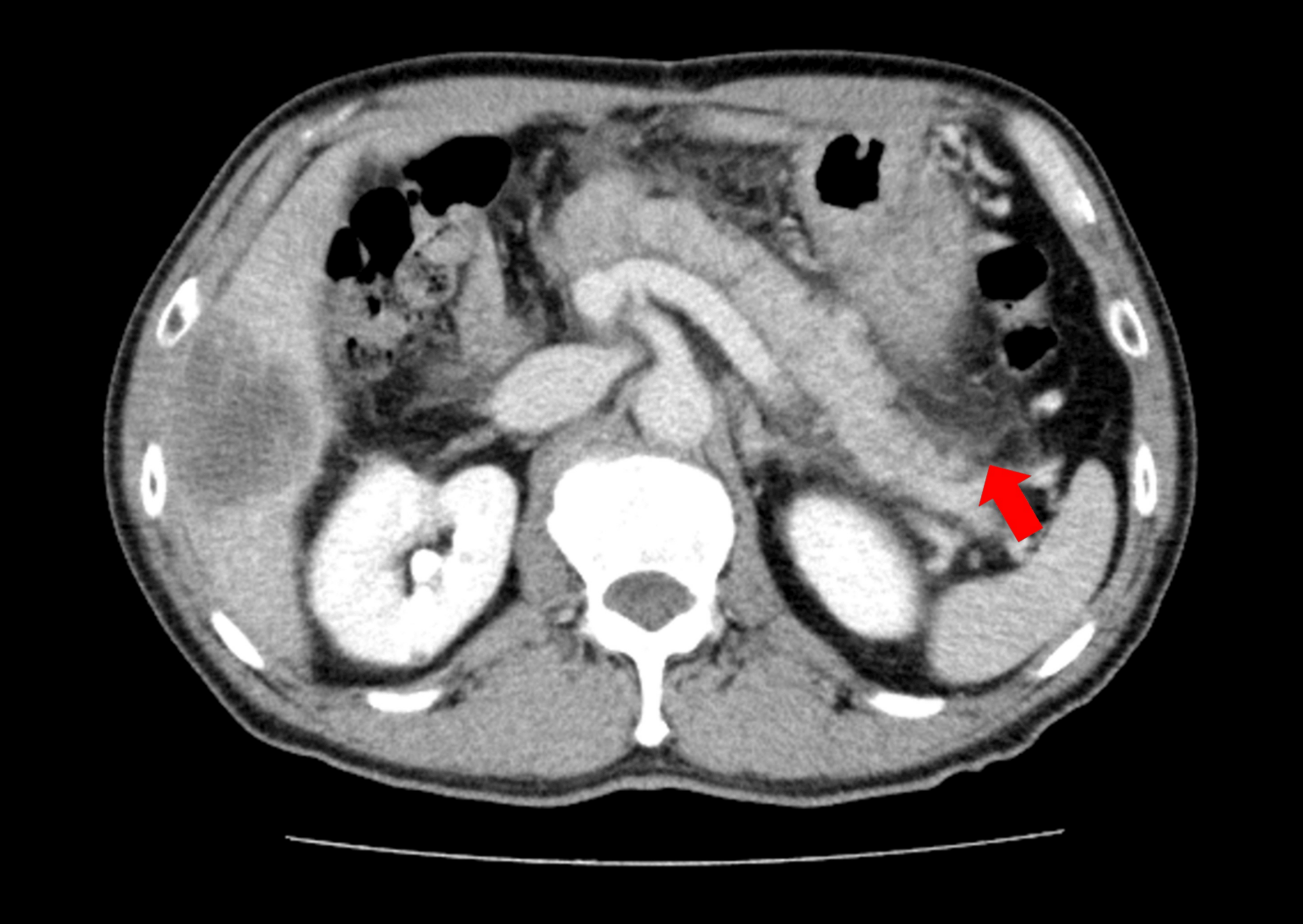
Follow-up CT of the remnant pancreas (April 2024) Contrast-enhanced abdominal CT taken in April 2024 shows persistent but stable inflammatory changes around the remnant pancreas (red arrow), with no new fluid collection or necrosis.

A second course of GnP therapy was administered on April 16, 2024, using the same antiemetic regimen. Several hours after treatment, the patient again developed epigastric pain and was diagnosed with recurrent mild acute pancreatitis (Figure 4). To exclude pancreaticojejunostomy stenosis and allow continuation of chemotherapy, pancreatic duct stent placement was performed on April 24, 2024.

Despite these interventions, a third course of GnP therapy administered on May 1, 2024, again resulted in acute pancreatitis on the same day. After multidisciplinary discussion and with informed consent, the chemotherapy regimen was changed to mFFX because of progressive metastatic disease.

mFFX therapy was initiated on May 10, 2024. Antiemetic prophylaxis consisted of intravenous palonosetron (0.75 mg) and dexamethasone (4.95 mg). On the same day, the patient developed mild epigastric discomfort with elevation of pancreatic enzymes, consistent with recurrent acute pancreatitis. Conservative management was effective.

Concomitant medications during this period included tramadol/acetaminophen, lansoprazole, magnesium oxide, and pancrelipase. These medications had been used continuously before and after the episodes of pancreatitis without a temporal association with symptom onset. Further anticancer treatment was not feasible because of disease progression, and the patient died in July 2024.

## Discussion

Drug-induced acute pancreatitis is relatively uncommon, accounting for approximately 0.1-2% of all cases, and is generally regarded as a diagnosis of exclusion [2,3]. In Japan, alcohol consumption and gallstones remain the most common etiologies of acute pancreatitis [1]. However, with increasing use of combination chemotherapy and supportive care medications, the spectrum of drugs potentially associated with pancreatic injury has expanded. Previous narrative reviews and systematic reviews emphasize that, before diagnosing drug-induced acute pancreatitis, common alternative etiologies, including gallstones, alcohol consumption, hypercalcemia, hypertriglyceridemia, biliary disease, infection, trauma, and anatomical abnormalities of the pancreas, should be carefully considered and excluded as appropriate [2,9]. In the present case, these alternative causes were systematically evaluated and considered unlikely.

A notable feature of this case was the repeated onset of acute pancreatitis within 24 hours after initiation of both GnP and mFFX therapy. Furthermore, pancreatitis recurred despite endoscopic evaluation and intervention for the pancreaticojejunostomy, suggesting that mechanical obstruction was unlikely to be the primary cause. This short latency and reproducible clinical course strongly support a drug-related mechanism.

Identification of a single causative agent was challenging because multiple anticancer and supportive medications were administered concurrently. Gemcitabine and nab-paclitaxel are widely used, and acute pancreatitis is not recognized as a common adverse effect [5]. Although acute pancreatitis has been reported with 5-fluorouracil, evidence supporting a definitive causal relationship remains limited. In addition, chronically administered concomitant medications were considered less likely contributors because they were administered without dose changes and showed no consistent temporal relationship to the acute episodes, whereas pancreatitis reproducibly occurred within hours after chemotherapy administration with antiemetic premedication; however, they cannot be completely excluded, given the variable latency of drug-induced pancreatitis.

Medications common to both regimens included 5-hydroxytryptamine type 3 (5-HT3) receptor antagonists and corticosteroids. Corticosteroids have previously been reported in association with acute pancreatitis; however, critical reviews suggest they are unlikely to be causative in most cases [3]. In contrast, sporadic case reports have described acute pancreatitis temporally associated with 5-HT3 receptor antagonists such as granisetron [9,10]. Nevertheless, these reports are limited to individual cases, and the overall quality of evidence is low. Notably, acute pancreatitis recurred despite switching the 5-HT3 receptor antagonist from granisetron during GnP therapy to palonosetron during mFFX therapy, suggesting a potential class-effect adverse reaction rather than an agent-specific effect. In this patient, pancreatitis occurred in the remnant pancreas after pancreaticoduodenectomy, a condition characterized by altered anatomy due to pancreaticojejunostomy. Importantly, anastomotic stenosis was ruled out by endoscopic evaluation, supporting the interpretation that the pancreatitis was drug-induced rather than mechanically mediated.

This interpretation is consistent with systematic reviews of drug-induced acute pancreatitis, which indicate that most drug-pancreatitis associations are derived from case reports and retrospective data, precluding definitive causal conclusions [9]. Causality was further assessed using the Naranjo Adverse Drug Reaction Probability Scale [11]. The GnP regimen yielded a total score of 6 (probable), based on temporal association, improvement after withdrawal, and recurrence upon re-exposure. The mFFX regimen yielded a total score of 4 (possible), reflecting temporal association and improvement after discontinuation, although reproducibility could not be fully confirmed. Several items were not scored because of the combination chemotherapy and overlapping supportive medications.

Taken together, this case illustrates the complexity of evaluating acute pancreatitis in patients receiving combination chemotherapy. The close temporal association and recurrence across different regimens support a drug-related mechanism, while the presence of multiple agents and patient-specific factors preclude definitive identification of a single causative drug.

## Conclusions

In conclusion, this case suggests that shared medications, including supportive care agents, may have contributed to recurrent acute pancreatitis during combination chemotherapy.

Clinicians should remain vigilant regarding drug-induced pancreatitis caused not only by cytotoxic agents but also by supportive care medications, such as antiemetics, especially in patients with a history of pancreatic resection.
